# Myalgia-arthralgia syndrome induced by docetaxel in oncology: the wolf disguised as a sheep

**DOI:** 10.1590/1516-3180.2018.0046180218

**Published:** 2018-06-18

**Authors:** Esther Una Cidon

**Affiliations:** I MD, MSc, PhD. Medical Oncologist, Department of Medical Oncology, Royal Bournemouth and Christchurch Hospital NHS Foundation Trust, Bournemouth, United Kingdom.

Taxanes are anti-microtubulin drugs that have a broad spectrum of antitumor activity. They are probably the most widely used cytotoxics.^1^ Their common adverse effects include bone marrow suppression, hypersensitivity or skin reactions, alopecia and peripheral sensory neuropathy^1^. However, the myalgia-arthralgia syndrome (MAS), which is frequent in daily practice, remains the great unknown. There are no data regarding its incidence or appropriate treatment and this is a matter for concern, since it impacts negatively on patients’ compliance.

MAS usually appears within the first hours or days after taxane infusion and patients complain of moderate to severe generalized musculoskeletal pain: sometimes comprising joint and bone pains and at other times neuropathic pain, with sensations of electrical shock and weakness in both legs.^1^

Recently, professionals have become more aware of the relevance of MAS, and this is probably because most protocols in cases of early breast cancer use a sequential regimen with fluorouracil, epirubicin and cyclophosphamide (FEC) followed by docetaxel monotherapy at a dose of 100 mg/m^2^, as opposed to the dose in combination.^2^ A recent review with 102 patients^1^ reported that around 30% of them suffered from MAS, but did not give definite answers as to why this syndrome is developed or how to treat it adequately. Extended doses of steroids, pregabalin, gabapentin, non-steroidal anti-inflammatories, opioids, etc., have been tried with contradictory results.^3^ Unfortunately, MAS has been the most relevant reason for reducing the dose or definitively halting the oncological treatment, and this may place survival outcomes at risk.

We reviewed 100 patients with localized breast cancer treated during 2015 and 2016 in our Department. These received docetaxel at a dose of 100 mg/m^2^. We collected data about the incidence of MAS, rate of discontinuation and dose reduction due to this syndrome. Briefly, 84 patients developed MAS after the first cycle, which was a much higher incidence than expected. They complained of generalized pain and sometimes “shooting pains” in both legs, usually starting 3 or 4 days after the infusion and lasting on average 7 days (range: 4-10 days). Most of these patients had grade 1 or 2 symptoms, but 22% had grade 3 and they needed a dose reduction. Some of them asked for interruption (2%). Initially, those patients were treated with non-steroidal anti-inflammatories, paracetamol and steroids, but none of these helped. Then we decided to try a combination of codeine and paracetamol, or morphine if the patients were intolerant to codeine. With this, the figures changed completely. No further dose modifications were needed, and the patients coped well with the treatment (MAS grade 0-1, but manageable).

In 2018, we completed a prospective study at our department, in which 60 patients receiving docetaxel at 100 mg/m^2^ were included. All received a combination of codeine and paracetamol and the instructions to use it immediately if symptoms of MAS appeared. Patients had received adequate education with explanations about what to expect, when and how to treat it immediately. We noted that there was no dose reduction of docetaxel or interruption due to MAS. Our results are very encouraging, because the patients have been able to complete their oncological treatment as planned. We consider it important to share these results with all professionals, and not only oncologists, since many of these patients seek help in the emergency departments when they become very worried about this generally forgotten adverse event.
